# Organizing graduate medical education programs into communities of practice

**DOI:** 10.3402/meo.v21.31864

**Published:** 2016-10-05

**Authors:** Robert G. Bing-You, Kalli Varaklis

**Affiliations:** Department of Medical Education, Maine Medical Center, Portland, ME, USA

**Keywords:** medical setting, organizational culture, staff management, residency

## Abstract

**Background:**

A new organizational model of educational administrative support was instituted in the Department of Medical Education (DME) to better meet increasing national accreditation demands. Residency and fellowship programs were organized into four ‘Communities of Practice’ (CoOPs) based on discipline similarity, number of learners, and geographic location. Program coordinator reporting lines were shifted from individual departments to a centralized reporting structure within the DME. The goal of this project was to assess the impact on those most affected by the change.

**Methods:**

This was a mixed methods study that utilized structured interviews and the Organizational Culture Assessment Instrument (OCAI). Eleven members of the newly formed CoOPs participated in the study.

**Results:**

Three major themes emerged after review and coding of the interview transcripts: improved group identity, improved availability of resources, and increased opportunity for professional growth. OCAI results indicated that respondents are committed to the DME and perceived the culture to be empowering. The ‘preferred culture’ was very similar to the culture at the time of the study, with some indication that DME employees are ready for more creativity and innovation in the future.

**Conclusion:**

Reorganization within the DME of residency programs into CoOPs was overwhelmingly perceived as a positive change. Improved resources and accountability may position our DME to better handle the increasing complexity of graduate medical education.

The advent of the Next Accreditation System (NAS) (1) by the Accreditation Council for Graduate Medical Education (ACGME) prompted our medical education leaders to conclude that a different organizational model of educational administrative support was required within the central Department of Medical Education (DME) to better meet the demands of the NAS. The NAS created new accreditation challenges (e.g., more frequent collection, analysis, and reporting of data) for graduate medical education (GME) programs and there was collective agreement that increased collaboration between programs was required to improve program performance.

‘Communities of Practice’ (CoOP) have been defined as ‘groups of people informally bound together by shared expertise and passion for a joint enterprise’ (2), and have the potential to stimulate knowledge sharing, learning, and change. We organized the administrative staff and program directors supporting GME programs (i.e., both residencies and fellowships) into four CoOPs based on discipline similarity, number of learners, and geographic location: surgery, hospital-based, primary care, and internal medicine CoOPs. All administrative staff within the CoOPs would now directly report to the centralized DME, instead of functioning within individual departmental silos with variable reporting lines. Such administrative staff are commonly known as ‘program coordinators’ and have typical responsibilities such as being involved with all aspects of the annual recruitment process, disseminating and collecting resident and program evaluation data, scheduling of educational rotations and conferences, and supporting the program director. Prior to the reorganization, individual staff members could meet with each other on an informal, *ad hoc* self-driven basis. Individual administrative staff often had competing roles and responsibilities (e.g., scheduling patients), had no opportunities for promotion within an educational system, and had variable or absent performance reviews.

Goals of the reorganization also included restructured job descriptions such that GME staff members were dedicated to GME full-time, to improve the ability of the DME to provide professional development to staff members, and to create a cadre of dedicated educational support personnel to improve succession planning. Each staff member would continue to support predominantly one to two GME programs and its residency program director, and remain in their current physical location to maintain accessibility to the residents. The 3–4 staff members within each CoOP were allowed to self-organize and establish how they would function as a community (e.g., frequency of meetings).

Assessing the organizational impact of creating CoOPs was the focus of this pilot study after the formation of the first two CoOPs. A well-studied framework for assessing organizational changes was selected to survey participants. The ‘Competing Values Framework’ (CVF) (3) identifies how individuals view organizations within a mixture of four frames: the Clan climate, the Hierarchy climate, the Adhocracy climate, and the Market climate. Developed over two decades ago, the CVF (4) differentiates two dimensions of organizational culture: one focused on flexibility and discretion versus stability and control, and the other focused an internal versus external orientation. These two dimensions form four quadrants when the results are plotted graphically, with each quadrant representing the four distinct frames. The Clan climate reflects a sociable working environment, where individuals view it as a ‘family.’ The Hierarchy climate is more focused on a formal and structured work environment, where keeping the organization functioning efficiently is important. The Adhocracy climate is one where individuals take risks in an energetic, innovative, and creative environment. The Market climate is more results-based and individuals tend to concentrate on completing goals in a competitive manner. Knowledge of a group's predilection could be useful (e.g., specific leadership tools or group activities would be selected). We chose the CVF model in order to assess how staff members perceived the current organizational climate as well as their preferred climate because we theorized the perceived organizational culture could impact the transition to CoOPs.

## Method

### Design

This was a mixed methods study that utilized semi-structured interviews and a survey instrument to elicit the perspective of GME administrative staff members and program leaders after the reorganization. We chose a convergent parallel design (5) as we had planned to collect and analyze the quantitative and qualitative data at one time, and we believed the data would be complementary to one another. The research project was determined to be exempt research by the Maine Medical Center Institutional Review Board.

### Setting and participants

The study took place at Maine Medical Center, Portland, Maine, which is an independent, academic medical center with 25 ACGME-accredited GME programs and one non-accredited fellowship. Two CoOPs were initially formed. All nine GME administrative staff members from the first two formed CoOPs were recruited for this project: 1) hospital-based CoOP (Radiology, Psychiatry, Child Psychiatry, Emergency Medicine) and 2) surgical CoOP (Anesthesiology, Obstetrics and Gynecology, General Surgery, Vascular Surgery, Urology). Additionally, two residency program directors and two department Chairs from departments within these two CoOPs were invited to participate.

### Interview guide and procedure

Semi-structured, in-depth interviews (Table 1) were conducted individually with each of the 13 participants to explore perspectives on the new administrative reorganization into CoOPs, which had occurred 1 year earlier. Interviews lasted approximately 45 min and were audiotaped. Interviews were conducted in June 2015, by a project manager in the DME who is not directly involved with GME and who has experience in qualitative research.

**Table 1:** Interview guide

How does your Community of Practice help you get things done now, compared to before you were in a Community of Practice?
What are the factors that facilitate making things happen in your Community? In your residency program? At ________ Center?
When things are not moving along as you would like, how do you figure out what to do?
What does it feel like to be in a Community now?
What does it mean to you to be in a Community?
How do you relate to the other administrative staff and get along with one another in your Community?
How about relating to those in your department, such as the residents, Program Director, and Chair?
Describe successes to date.
Describe challenges to date.
How would you describe your Community to someone who was interested in joining your Community, for example, someone applying for an administrative staff position in your Community?
What stories would you tell this applicant about your Community of Practice?
What does being in the Community mean for your job growth, or career plans?
What else would you like to say about your Community of Practice?

At the conclusion of each interview, participants were asked to complete the Organizational Culture Assessment Instrument (OCAI) (4), which was completed and collected anonymously on paper. The audiotapes were transcribed verbatim and the text uploaded into a qualitative research software system (NVivo10) for coding, review, and analysis.

### Data analysis

Following a convergent parallel design approach (5), the quantitative and qualitative data were analyzed separately. Thereafter, the two sets of data were merged and interpreted.

A grounded theory approach was chosen for the semi-structured interviews because an inductive analysis of the data was thought to be more appropriate than a deductive imposition of any general theory. Grounded theory is a method ‘that calls for a continual interplay between data collection and analysis to produce a theory during the research process.’ (6). Two transcripts were independently reviewed by the two investigators to develop an initial coding scheme. A common coding theme was agreed upon and utilized by both investigators to independently index and map themes in all 11 transcripts (nine administrators and four physician GME leaders), using the constant comparative method (7). Both investigators agreed after review of all transcripts that data saturation had been achieved (i.e., no new additional information was forthcoming and further coding was not needed) and no additional interviews were necessary.

**Fig. 1:**
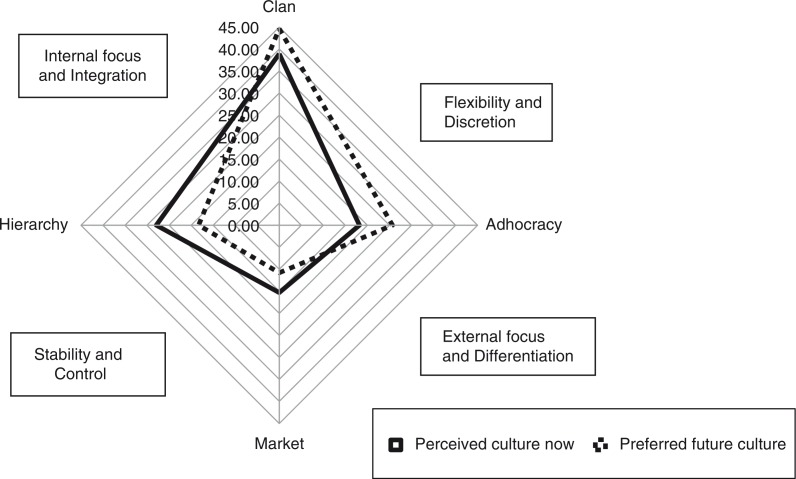
Organizational culture assessment polygons indicating current perceived culture now (after reorganization) and preferred future culture (means of all individuals scores, *n*=10).

The OCAI (4) consists of six questions, each question having four alternatives. Individuals are asked to divide 100 points between the four alternatives based on their perceived extent that each alternative is similar to their own organization. The first pass through the six questions is based on ‘now’ and the second pass is based on ‘preferred.’ An individual's scores are then averaged across the six questions, resulting in an average score for each of the four cultures (i.e., Clan, Adhocracy, Market, and Hierarchy), which are then plotted on the X- and Y-axis as shown in Fig. 1.

## Results

### Interviews

Seven GME administrative staff members from different departments within the Surgical and Hospital-based CoOPs agreed to participate in the study; one declined citing ‘no interest in participating in the study’ and one declined to find 45 min of time within the study period to schedule the interview. All staff members had worked in their current jobs from 1 to 35 years (mean 12.1 years, median 5 years). The four physician GME leaders also agreed to participate. All physicians had been in their current GME leadership positions from 1 to 22 years (mean 10.5 years, median 9.5 years). The interviewers asked all of the questions of all participants except one chair, who did not answer the last four questions of the structured interview guide and did not complete the OCAI survey due to time constraints.

After review and coding of the interview transcripts, three major themes emerged: 1) group identity, 2) improved availability of resources and help, and 3) professional growth opportunities.

### Group identity

The consistent theme expressed by all administrative staff was a renewed and positive group identity within the newly formed CoOPs. The initials associated with the quotes below represent pseudonyms for the participants.

I know that if I need something I can quickly go down the hall and they're going to help me out, and I know [ ] feels, as a new person, that she can call upon any of us, and it's just a very cohesive group. 1B

Although communication was cited as a strength of the new communities, there is no baseline for comparison because there was no expectation of communication amongst these individuals before the reorganization. In addition, the interview guide did not specifically ask for communication before and after the reorganization.

I think that sentiment is overused and over expressed, ‘communication is really important,’ but it's incredible important! We meet religiously every week. We have a team meeting every week, and if our regular meeting time doesn't work for one or more members, we move it to another time that week. We're committed. 1C

### Availability of resources

Another prominent theme expressed by most of the participants was the increased availability of resources and support for administrative staff within the community of practice.

Before, when you were going crazy, you just had to do it and there was nobody to help whereas now I feel like if I was really ‘I'm swamped! I need help!’ then people would say, ‘Ok. What do you need?’ 1F

I am supported by a community of people who do jobs similar to mine who would understand and be able to help if there was anything that the residents needed when they were to come in and that our hopes are that we can be interchangeable with any issues that might happen when one of us is out and so that we work together as a group as opposed to separate entities. IB

The reorganization, not unexpectedly, caused concern and job insecurity within the community (e.g., would staff resources be eliminated?), particularly with the two most experienced GME administrative staff who shared that they had significant concerns about the reorganization prior to its execution:

I was extremely reluctant and apprehensive about the change because it was kind of mysterious, I couldn't envision how it was really going to play out … I was hoping I'd be wrong that I was so against the change. And I readily admit that this is much better than I thought this would be. 1C

… there was a lot of insecurity when they started to restructure all of the jobs. I know I felt that way, and they were talking about nobody losing their job or position and not being cut but you could clearly feel that they were bringing more people in and feel that there were more people than there were positions … I am feeling much more positive about it than I was when I was thinking about it in theory. 1B

More recently, hired administrative staff did not express similar concerns about the reorganization, and one remarked that one of the challenges and subsequent success of the reorganization was the resolution of the negative attitude about the reorganization from some of the administrative staff.

having some of the coordinators on the team who were negative and against the change, having them now be positive and willing to share that experience with others has been a success. 1A

### Professional growth opportunities

A final prominent theme was that of improved opportunities for administrative staff professional development in GME, particularly for the two lead staff members who were required to take leadership courses within the healthcare system to prepare them for their new leadership roles within the CoOPs (e.g., some staff members now had supervisory roles of other staff).

coming into this I knew I wanted to do more than the staff role so this has really helped me get this exposure to supervising and team leadership so that in itself has been wonderful. I feel like I have done a crash course on HR so for me it has been key professional development. 1H

### OCAI survey results

Ten participants (seven administrators and three physician GME leaders) agreed to complete the OCAI survey immediately after the interview. The means of all individual results are graphically represented in Fig. 1. The solid polygon (‘Perceived culture now’) in the graph is markedly on the top of the vertical axis, indicating that the predominant culture in the new organization is in the ‘Clan’ culture, which is typical of an internally focused organizational structure characterized by commitment and loyalty from employees, who are able to create a work environment that takes into account the priorities of the members (8). The dotted line polygon in the graph is shifted even further to the top of the vertical axis and indicates respondents preferred even more of a Clan culture in the future than the current state. The graph results are also more shifted to the upper right quadrant, indicating that employees are empowered to make decisions within the larger structure.

There is concordance between the polygons graphically representing the perceived culture of CoOPs, ‘now’, at the time of the study compared to the ‘preferred’ future culture. The results indicate that the preferred future culture is shifted even more into the ‘Flexibility and Discretion’ cultural dimension, suggesting that the preferred culture would be characterized by even more flexibility and empowerment of individuals to make decisions. The ‘preferred’ culture polygon also suggests that respondents envision a culture more in the adhocracy culture, characterized by creativity and innovation, suggesting that respondents may be ready to take more risks.

## Discussion

Our pilot project explored the impact of an organizational change on those most affected. By merging and interpreting the qualitative and quantitative data, our results add to the literature in suggesting that an administrative reorganization within a centralized DME, while associated with initial anxiety particularly in employees who have been employed for a longer time in the previous administrative structure, is associated with a strong perception of increased support and resources for GME administrative staff within the newly formed CoOPs. The model by Bolman and Deal (9, 10) has been studied extensively in various settings, including educational (11) and medical organizations (12, 13), and the model offers one tool for looking at organizations through multiple perspectives (i.e., frames). Within the model, individuals tend to perceive an organization in one of four frames: human resource, political, symbolic, and structural. The perceptions of our participants from the qualitative data align with the ‘structural frame’ within the Bolman and Deal's model (9), suggesting that the new organizational structure provides clear goals, an unambiguous task-directed organizational structure, and clear lines of authority (8). This refocused clarity to task and structure may position the centralized, institutional department to better handle the complexity of the ever-changing climate of GME, such as the NAS (1). Benefits of the CoOP organizational model such as sharing best practices recruiting talent (2) and professionalization of administrative positions may follow.

Improved group identity appeared to be a strong result of the reorganization within the DME, with all administrative staff expressing satisfaction with group identity within their new communities. There were recurrent references to improved cohesion within the individual groups, defined by Carron (14) as having a common identity, sense of shared purpose, and a structured pattern of communication. Group cohesiveness has been consistently associated with productivity; in work settings with high performance norms, high group cohesiveness is associated with increased productivity (15). These perceptions align well with the ‘Human Resources’ frame of Bolman and Deal's framework (9) in which the organizational structure is supportive of individual participation and ownership in a collaborative environment and is willing to empower employees to take ownership for improved output. This suggestion from the qualitative data is strengthened with the integration of the quantitative results of the OCAI survey, which are strongly partial to the ‘Clan’ model. The Clan model is consistent with an organizational structure that is internally focused and empowering of employees to make decisions within the larger context.

One of the strengths of this pilot study was limiting bias by selecting an interviewer who was unaffected by the organizational change. Recall bias was minimized by completing the project temporally close to the organizational change. The results of the study are limited by the fact that two of the administrative staff declined to participate in the project. Because there was no pressure to participate, these two individuals were not probed further to explore their reasons for not participating. However, even if their views on group identity and improved resources would have not been favorable, they would have still been in the minority, given the unanimous sentiments of the other administrative staff. An additional limitation is the variability of the experiences of the administrative staff, with two having been hired within a year of the organizational change. Another limitation is that both investigators (one having a high-level supervisory role) are within the DME. No measures to ensure data trustworthiness were gathered in this initial project. Although the project had a relatively small sample size, due to the relatively homogeneous participant pool, data saturation was determined to have occurred after 11 interviews. The applicability and scalability of the CoOP model to institutions with larger numbers of GME programs remain to be evaluated. It is also unclear whether similar outcomes would be found if interviews were conducted prior to the reorganization. These results and conclusions are limited to one institution and may vary at other institutions.

Besides describing what CoOPs are (2), Wenger et al. have further described the ‘landscapes of practice’ (16). Their use of a landscape metaphor highlights the importance of interactions and boundaries between CoOPs, and how individuals often have to handle multimembership in several CoOPs (e.g., identity work through engagement, imagination, and alignment). As a model for GME within an organization, CoOPs may be enhanced by ‘systems conveners’ actively facilitating ‘habits of interaction’ (e.g., doing work together, ensuring relevant participation) as bridges between CoOPs (16). As the other CoOPs are created, and as the entire DME CoOP grows, our attention going forward will need to be more broadly focused on the dynamic and evolving nature of this GME landscape.

## Conclusion

With the integration of two sources of data, our results indicated that a reorganization of GME administrative staff into CoOPs resulted in improved perception of group identity, availability of resources, increased professional development, and possibly improved communication. The characterization of these attributes appears to align with Bolman and Deal's ‘Structural’ and ‘Human Resources’ framework. The results of the OCAI survey suggest that, after reorganization, respondents preferred a culture more characterized by a creative, dynamic environment ready for innovation and challenge.

These new CoOPs are in the beginning stages of group development, as described by the Five-Stage Group-Development model (17), and require further cultivation (2). There will be future opportunities to examine the groups’ performance as they move more cohesively to the ‘norming’ and ‘performing’ stages, including the later-formed primary care and internal medicine CoOPs. Future research could be done in other institutions to explore CoOPs as a useful organizational model for GME programs, whether interinstitutional CoOPs could be envisioned, and the quality of work across the GME landscape of practice (16).
